# Poverty and Internalizing Symptoms: The Indirect Effect of Middle Childhood Poverty on Internalizing Symptoms via an Emotional Response Inhibition Pathway

**DOI:** 10.3389/fpsyg.2016.01242

**Published:** 2016-08-17

**Authors:** Christian G. Capistrano, Hannah Bianco, Pilyoung Kim

**Affiliations:** Department of Psychology, University of Denver, Denver, COUSA

**Keywords:** middle childhood, poverty, family income, emotional response inhibition, internalizing symptoms

## Abstract

Childhood poverty is a pervasive problem that can alter mental health outcomes. Children from impoverished circumstances are more likely than their middle-income counterparts to develop internalizing problems such as depression and anxiety. To date, however, the emotional-cognitive control processes that link childhood poverty and internalizing symptoms remain largely unexplored. Using the Emotion Go/NoGo paradigm, we examined the association between poverty and emotional response inhibition in middle childhood. We further examined the role of emotional response inhibition in the link between middle childhood poverty and internalizing symptoms. Lower income was associated with emotional response inhibition difficulties (indexed by greater false alarm rates in the context of task irrelevant angry and sad faces). Furthermore, emotional response inhibition deficits in the context of angry and sad distracters were further associated with child-report internalizing problems. The results of the current study demonstrate the significance of understanding the emotional-cognitive control vulnerabilities of children raised in poverty and their association with mental health outcomes.

## Introduction

Poverty is a pervasive social problem that affects millions of families in the USA. Currently, over 16 million children under the age of 18 live in households that fall below the poverty line (United States Census Bureau, 2013). One of the most consistent findings in the literature is the inverse relationship between childhood poverty and mental health (Tracy et al., 2008; Evans and Cassells, 2013). The current study focuses on internalizing symptoms (i.e., symptoms of anxiety and depression) that are frequently associated with childhood economic disadvantage (Brooks-Gunn and Duncan, 1997; Murali and Oyebode, 2004; Slopen et al., 2010). The prevalence of symptoms of depression and anxiety is greater among children from low-income backgrounds (Duncan et al., 1994; Gilman et al., 2002; Murali and Oyebode, 2004). To date, however, we have limited understanding of the emotional-cognitive processes by which poverty and these internalizing symptomatologies are associated. Behavioral and neuroimaging research in both high-risk and typically developing children suggests that deficits in emotional response inhibition (i.e., the ability to inhibit a behavioral response in the context of negatively valenced emotional distracters) are associated with greater levels of childhood anxiety and depression (Tottenham et al., 2010; Kilford et al., 2015). However, few studies have examined the influence of socioeconomic disadvantages such as poverty on emotional response inhibition, which can in turn increase risks for internalizing symptoms. Thus, the goals of the present study are: (1) to investigate the relationship between family income and emotional response inhibition in middle childhood and (2) to explore the indirect effect of childhood poverty on internalizing symptoms via an emotional response inhibition pathway.

Emotional response inhibition refers to one’s ability to inhibit a pre-potent response to emotionally charged stimuli (Schulz et al., 2007, 2009). In the current study, this specifically refers to the ability to inhibit responses to irrelevant emotional information. This may also be described as the tendency to orient attention to salient but irrelevant emotional information, and subsequent difficulties disengaging attention away from these emotional stimuli. Difficulties disengaging attention away from these emotional cues can in turn affect cognitive control processes or goal directed behavior (i.e., inhibiting a response to irrelevant information). Using the Emotion Go/NoGo paradigm – a task that measures response inhibition in the context of emotional distracters, studies have found that difficulties with emotional response inhibition in the context of negatively valenced emotional distracters (i.e., angry, sad, or fearful information) can be a risk factor for anxiety and depression (Tottenham et al., 2010; Kilford et al., 2015). The Emotion Go/NoGo requires participants to rapidly respond to a set of “Go” target neutral facial expressions presented with a series of “NoGo” distracter emotional facial expressions (happy, fearful, angry, and sad), or vice versa. The number of false responses to emotional NoGo distracters serves as a primary measure of emotional response inhibition (Schulz et al., 2007, 2009). Indeed, adolescents diagnosed with depression exhibit greater response inhibition difficulties in the context of negative emotional stimuli (Kilford et al., 2015), as indexed by greater false alarm rates to sad stimuli in a modified Emotion Go/NoGo task. Using the same paradigm, Tottenham et al. (2010) found that response inhibition difficulties in the context of negatively valenced facial expressions (averaged across fearful, angry, and sad faces) are associated with early adversity exposure (i.e., being previously institutionalized), amygdala hypertrophy, and anxiety symptoms in middle childhood.

As suggested by the Tottenham et al. (2010) study, being previously institutionalized is an important model of early adversity. However, it is an extreme and relatively atypical experience for most children. To date, there is little understanding of whether relatively typical examples of early adversity such as poverty may be associated with deficits in emotional response inhibition. However, existing literature provides consistent evidence of the negative effects of poverty on cognitive and emotional processes. Several neuroimaging studies have demonstrated the link between childhood socioeconomic disadvantage and anatomical and functional changes to brain regions involved in emotion processing of negative information (such as angry or fearful faces), including the amygdala (Gianaros et al., 2008; Hackman et al., 2010; Noble et al., 2012; Luby et al., 2013). In addition, childhood poverty has also been associated with altered structural and functional changes in brain regions that are related to difficulties in emotion regulation in response to negative social cues, including the prefrontal cortex (Kim et al., 2013; Javanbakht et al., 2015; Liberzon et al., 2015). These imaging findings are consistent with physiological and behavioral evidence suggesting heightened sensitivity to negative emotional information (Evans and Kim, 2007; Evans et al., 2013), and greater negative affect among poor children (Murali and Oyebode, 2004; Barajas et al., 2007; Tracy et al., 2008; Najman et al., 2010). Currently, however, less is known about how heightened sensitivity to negative emotional information interact with cognitive function. Childhood poverty exposure has been associated with inhibitory control difficulties (Spielberg et al., 2015), but whether such difficulties may be influenced by emotional context is largely unknown. Thus, the current study aims to examine how emotional sensitivity to negative emotional information among children who are exposed to poverty may disrupt inhibitory control. Understanding the links between poverty and emotional response inhibition would be significant because deficit in emotional response inhibition has been associated with internalizing problems in children and adults (Tottenham et al., 2010; Pacheco-Unguetti et al., 2012; Kilford et al., 2015).

In the present study, we investigate the role of emotional response inhibition in the relationship between family income and internalizing symptoms in middle childhood. Middle childhood is a particularly vulnerable developmental period when early symptoms of depression and anxiety emerge but largely predate clinical diagnosis (Costello et al., 2005; Thapar et al., 2012). Thus, understanding the associations between family income and emotional response inhibition processes at this developmental stage may have implications for prevention efforts before symptoms fully develop into clinical disorders during adolescence. We hypothesize that family income will be negatively associated with internalizing symptoms. We also hypothesize that family income will be negatively associated with emotional response inhibition, such that children from lower-income households will exhibit response inhibition problems (i.e., greater false alarm rates) in the context of task irrelevant negative facial expressions. We further hypothesize that emotional response inhibition problems among poor children will be further associated with greater internalizing problems.

## Materials and Methods

### Participants

Children and their biological mothers were recruited from the University of Denver’s volunteer database and through flyer distribution around Denver metro area public schools and anti-poverty programs. Interested participants contacted the research team and were screened for eligibility. Only one child per household was eligible to participate. The study targeted low-income and middle-income children (0 ≤ income-to-needs ratio ≤ 5; see Family Income). Children who were currently being treated for psychiatric disorders, had a past or present neurological diagnosis, or an IQ below 80 were excluded. One hundred forty-nine mothers completed the initial eligibility survey. Of those, 47 children and their mothers passed the initial screening and participated in the home visit. Two of the children did not complete the Emotion Go/NoGo computer task due to timing issues during the home visit, and two more were excluded after more detailed data collected during the home visit revealed participant ineligibility (i.e., having an IQ below 80 and a family income-to-needs ratio above 5). The final sample included 43 children (60% female; mean age = 8.7 ± 0.67; 56% White/Caucasian, 28% Black/African American, 9% Hispanic, 7% Bi-racial; **Table 1**).

**Table 1:** Characteristics of the participants.

	*N* (%)	Mean ± SD	Range
Child age (years)		8.70 ± 0.67	8–10
Child race/ethnicity			
White/Caucasian	24(56)		
Black/African–American	12(28)		
Hispanic	4(9)		
Bi-racial	3(7)		
Income-to-needs ratio		2.13 ± 1.37	0.00–4.95
IQ		100.84 ± 11.81	80–126
Child-Reported Internalizing scores (cSDQ)		3.37 ± 2.54	0–10
Child-Reported Internalizing scores (CBCL)		6.02 ± 5.59	0–23

### Procedures

A pair of trained researchers independently worked with the child participant and his/her mother and administered all measures in the participants’ home. Mothers provided written consent prior to participation. Children provided written and verbal assent in accordance with the University of Denver’s Institutional Review Board guidelines. Income-to-needs ratio and demographic information were collected during a structured interview with the child’s biological mother. IQ was assessed using the Wechsler Abbreviated Scale of Intelligence (WASI; Wechsler, 1999) by a trained researcher. IQ scores were calculated by summing the vocabulary and matrix reasoning subscale scores of the WASI. The Emotion Go/NoGo behavioral task was administered to the child using a laptop. Both mother and child completed a set of questionnaires about the target child’s internalizing symptoms.

### Measures

#### Family Income

Family income was assessed based on the family’s income-to-needs ratio. In absolute terms, poverty is defined as having an income level that falls below an established federal poverty threshold, and is a condition whereby the amount of family income does not meet basic needs such as food and shelter (United States Census Bureau, 2013). The ratio of family income-to-needs was computed by dividing the total parent-reported family income by the federal poverty threshold, adjusted for the number of individuals in the home. Family income-to-needs has been used as a primary measure of absolute poverty in a number of studies that examined poverty and socio-emotional development (Evans et al., 2005; Barajas et al., 2007; Slopen et al., 2010; Kim et al., 2013; Barch et al., 2015; Javanbakht et al., 2015; Liberzon et al., 2015; Raver et al., 2015). The income-to-needs ratio was calculated based on the parent-reported family income within the last 12 months from the date of the home visit (*M* = 2.15; *SD* = 1.35; Range: 0.00–4.95). Forty-nine percent of the sample lived in poverty (income-to-needs ratio ≤ 1) or near poverty (income-to-needs ratio ≤ 2).

#### Behavioral Task

**FIGURE 1:**
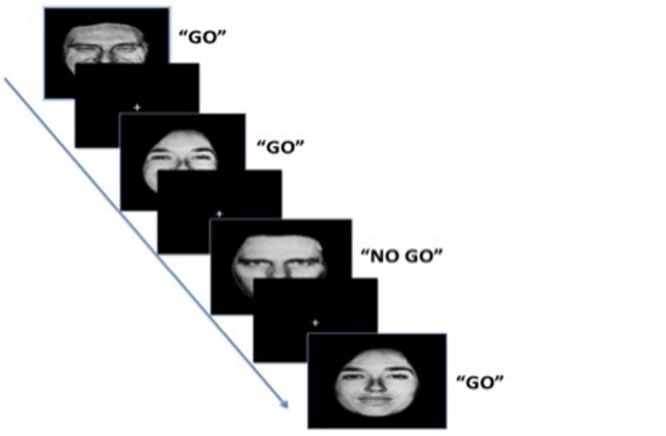
**Emotion Go/NoGo Task.** The trials were 70% Go targets. Each stimulus was presented for 500 ms with an interstimulus interval of 1000 ms.

The Emotion Go/NoGo task is an emotional response inhibition paradigm used in a similar age group (ages 5–12; Tottenham et al., 2011a,b). The Emotion Go/NoGo was administered on a laptop and took approximately 10 min to complete (Tottenham et al., 2010; task downloaded from the Sackler Institute Assays and Tools, 2014). Participants were asked to rapidly respond to a series of target facial expression (e.g., neutral, “Go” target trials) while inhibiting a response to a series of distracter emotional facial expressions (e.g., happy, fearful, angry, and sad, “NoGo” distracter trials), or vice versa (**Figure 1**). Neutral faces were always paired with either a positively valenced (i.e., happy) face or a negatively valenced (i.e., fearful, angry, or sad) face. There were a total of eight blocks (neutral/happy, neutral/fearful, neutral/sad, neutral/angry, happy/neutral, fearful/neutral, sad/neutral, angry/neutral) with 30 trials each. The trials were 70% Go targets to build a prepotent response. Each stimulus was presented for 500 ms with an interstimulus interval of 1000 ms. Stimulus presentation and inter-stimulus interval duration are considered to be appropriate for this age range based on children’s overall accuracy levels (Tottenham et al., 2011b). Stimulus presentation was randomized across conditions. Prior to the task, practice trials were administered to ensure that all participants fully understand the task. False alarms to NoGo emotional distracters were used as a primary measure of emotional response inhibition (Schulz et al., 2007). False alarm rates were defined as false responses to distracter stimuli and were calculated by dividing the number of false presses by the number of distracter stimuli. Reaction time (RT) and percentage of correct responses were calculated to examine overall task performance. The percentage of correct responses was calculated by summing false alarms and missed target responses then subtracting them from the total number of trials. RTs were calculated for correct trials only.

#### Internalizing Questionnaires

Internalizing symptoms were assessed by both self-report and mother-report questionnaires.

(A) Self-report: children completed the Strengths and Difficulties Questionnaire child-report version (cSDQ; Goodman and Scott, 1999). Large national studies have used the emotion subscale of the cSDQ as a self-report measure of emotional health and internalizing symptoms in middle childhood (age 6–10 years-old; Muris et al., 2003; Curvis et al., 2013). The emotion subscale consists of five questions (e.g., “I worry a lot”) scored on a three-point Likert scale: 0 = Not True, 1 = Somewhat True, 2 = Certainly True. Child-report SDQ has a good and comparable reliability and validity similar to a parent-report SDQ and Child Behavior Checklist (Goodman, 2001; Muris et al., 2003). Within our sample, Cronbach’s alpha for the five-item subscale is 0.70, which is comparable to data using large samples (Goodman, 2001; Giannakopoulos et al., 2013).

(B) Mother-report: mothers completed the Child Behavior Checklist (CBCL; Achenbach, 1991). The CBCL is a reliable and well-validated parent-report checklist that measures internalizing and externalizing behaviors (Nakamura et al., 2009). The internalizing subscale of the CBCL consists of 26 items (e.g., child’s “Feelings easily hurt?”) scored on a three-point Likert scale: 0 = Not True, 1 = Somewhat True or Sometimes, 2 = Very True or Often True.

### Analyses Plan

Percentage of correct responses and RT duration were examined for outliers (RT < 100 ms and > 1000 ms; Ladoucer et al., 2006), but no outliers were detected. All participants had above 50% accuracy (based on overall performance, averaged across Go and NoGo trials). Pearson correlations were conducted to examine the associations among variables. Demographic variables that were significantly correlated with family income were included as covariates.

A three-way repeated measures ANCOVA was conducted for the dependent measure of false alarm rate, with Family Income as a between-subject factor, and Emotion (happy, fearful, angry, and sad) and Condition (Go and NoGo) as within-subjects factors, with covariates IQ, race, and accuracy to “Go” trials. Significant interactions were decomposed using *post hoc* ANCOVA and regression analyses. RT and percentage of correct responses were added to the repeated measures ANCOVA with Family Income as a between-subject factor, and Emotion (happy, fearful, angry, and sad) and Condition (Go and NoGo) as within-subjects factors, with covariates. SPSS Version 23 was used for all interaction and *post hoc* analyses. To test the indirect effect of poverty on internalizing symptoms through an emotional response inhibition pathway, Preacher and Hayes’ (2004) bootstrapping method was used (mediation model number 4; 10,000 bootstrap resamples), which is an appropriate test of indirect effect in studies with small sample sizes. An indirect path is statistically significant if the associated 95% confidence interval (CI; bias corrected) does not include zero.

### Correlations among Variables

Family income was significantly correlated with the emotional subscale score of the cSDQ (**Table 2**), *r*(41) = -0.31, *p* < 0.05. While lower income was associated with greater levels of child-report internalizing symptoms, it was not significantly correlated with parent-report of internalizing symptoms on the CBCL. Race and IQ were significantly correlated with family income and were included as covariates in the main interaction analyses.

**Table 2:** Bivariate correlations between family income, child internalizing symptoms, and demographic variables.

	1	2	3	4	5	6	7
(1) Family income							
(2) Child reported internalizing symptoms	-0.311*						
(3) Mother reported internalizing symptoms	-0.243	0.297					
(4) IQ	0.458**	-0.473**	-0.064				
(5) Race/ethnicity (1 = White, 2 = Other)	-0.396**	0.390**	0.123	-0.413**			
(6) Sex	0.033	-0.013	-0.212	-0.150	-0.143		
(7) Age	0.215	-0.072	0.021	0.107	0.052	-0.010	

*p < 0.05; **p < 0.01.

Behavioral outcomes (false alarm rate, overall percentage of correct responses, RT) of the Emotion Go/NoGo task are included in **Table 3**. Overall mean accuracy, false alarm rates, and RTs are similar to studies of children within this age range (Tottenham et al., 2010, 2011b). ANOVA analysis examining the effects of Condition and Emotion yielded similar results to a previous study using the same task among children of comparable age range (Tottenham et al., 2011a; see more detailed results in Supplementary Materials).

**Table 3:** Behavioral results from the Emotion Go/NoGo task.

	Condition	Mean (SD)
Response time in milliseconds (range)	Happy targets (Go) in the context of Neutral distracters (NoGo)	472.32 ± 95.75 (314.50–756.29)
	Fear targets (Go) in the context of Neutral distracters (NoGo)	492.69 ± 118.75 (312.80–922.89)
	Angry targets (Go) in the context of Neutral distracters (NoGo)	502.85 ± 132.52 (295.23–891.76)
	Sad targets (Go) in the context of Neutral distracters (NoGo)	519.46 ± 130.93 (302.00–940.71)
		
	Neutral (Go) in the context of Happy distracters (NoGo)	496.08 ± 94.98 (337.09–747.15)
	Neutral (Go) in the context of Fear distracters (NoGo)	490.85 ± 111.15 (267.11–838.20)
	Neutral (Go) in the context of Angry distracters (NoGo)	503.82 ± 125.66 (297.79–928.74)
	Neutral (Go) in the context of Sad distracters (NoGo)	505.75 ± 137.93 (253.53–884.71)
		
False alarm (percentage) (Range)	Happy targets (Go) in the context of Neutral distracters (NoGo)	29.30 ± 22.72 (00–90)
	Fear targets (Go) in the context of Neutral distracters (NoGo)	36.98 ± 25.31 (0–100)
	Angry targets (Go) in the context of Neutral distracters (NoGo)	35.11 ± 22.72 (0–90)
	Sad targets (Go) in the context of Neutral distracters (NoGo)	50.23 ± 25.12 (10–100)
		
	Neutral (Go) in the context of Happy distracters (NoGo)	32.79 ± 19.68 (00–70)
	Neutral (Go) in the context of Fear distracters (NoGo)	36.98 ± 19.70 (00–90)
	Neutral (Go) in the context of Angry distracters (NoGo)	45.12 ± 23.74 (00–100)
	Neutral (Go) in the context of Sad distracters (NoGo)	56.74 ± 23.78 (00–100)
		
Percentage of Correct Responses	Happy targets (Go) in the context of Neutral distracters (NoGo)	84.19 ± 10.00 (56.67–96.67)
(Range)	Fear targets (Go) in the context of Neutral distracters (NoGo)	79.07 ± 12.20 (53.33–96.67)
	Angry targets (Go) in the context of Neutral distracters (NoGo)	76.05 ± 10.11 (53.33–100)
	Sad targets (Go) in the context of Neutral distracters (NoGo)	69.84 ± 11.22 (46.67–90)
		
	Neutral (Go) in the context of Happy distracters (NoGo)	81.78 ± 10.92 (43.33–100)
	Neutral (Go) in the context of Fear distracters (NoGo)	76.28 ± 10.82 (43.33–93.33)
	Neutral (Go) in the context of Angry distracters (NoGo)	74.81 ± 11.98 (43.33–96.67)
	Neutral (Go) in the context of Sad distracters (NoGo)	70.31 ± 10.18 (43.33–90)

### Emotional Response Inhibition

**FIGURE 2:**
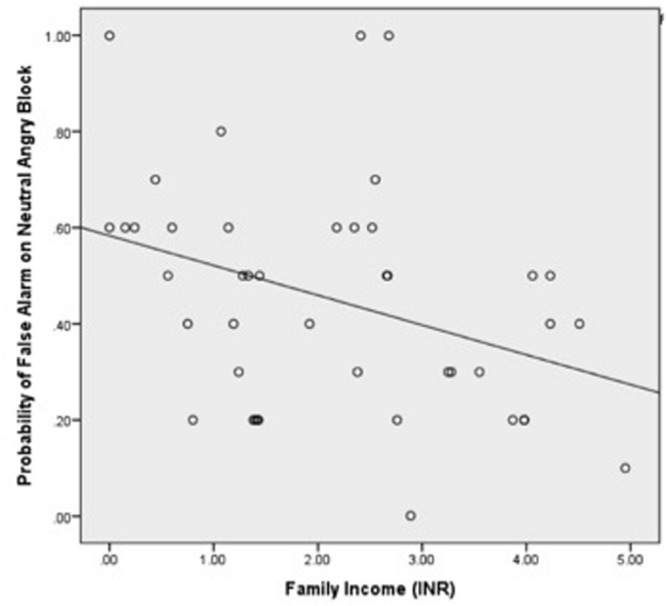
**Relationship between family income and the probability of false alarm rates during Angry “NoGo” distracter trials, *b* = -0.36, *p* < 0.05**.

**FIGURE 3:**
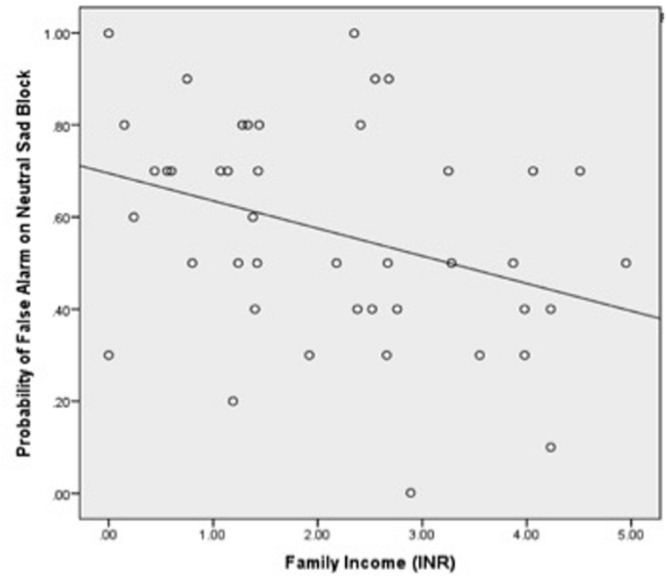
**Relationship between family income and the probability of false alarm rates during Sad “NoGo” distracter trials, *b* = -0.34, *p* < 0.05**.

We examined the association between false alarm rate and family income. A three-way Emotion × Condition × Family Income interaction on the dependent variable of false alarm rate was significant, *F*(3,93) = 4.14, *p* < 0.01, $\eta_{p}^{2}$ = 0.12. *Post hoc* analyses revealed a two-way Emotion × Family Income interaction in the NoGo condition only (when neutral target “Go” faces were presented with a series of emotional distracter “NoGo” faces), *F*(3,93) = 5.70, *p* < 0.005, $\eta_{p}^{2}$ = 0.16. *Post hoc* regressions revealed that lower family income was associated with higher false alarm rates during angry distracter face trials, *b* = -0.36, *p* < 0.05 (**Figure 2**), and sad distracter face trials, *b* = -0.34, *p* < 0.05 (**Figure 3**). No significant associations were found between family income and false alarm rates during fearful or happy distracter face trials. There were no significant interactions or main effects of Condition or Emotion with family income or a main effect of family income on the dependent variable of false alarm rate.

### Overall Performance

#### Percentage of Correct Responses

A three-way Emotion × Condition × Family Income interaction (with the percentage of correct responses as a dependent variable) was significant, *F*(3,117) = 2.68, *p* < 0.05, $\eta_{p}^{2}$ = 0.06. *Post hoc* ANCOVA revealed a two-way Emotion × Family Income interaction in the “Go” condition only (when emotional faces were the target stimuli), *F*(3,117) = 3.16, *p* < 0.05, $\eta_{p}^{2}$ = 0.08. *Post hoc* regression revealed that lower family income was significantly associated with lower percentage of correct responses during fearful target face trials (or when fearful faces were the “Go” target stimuli), *b* = 0.39, *p* < 0.01. There were no significant interactions of Condition or Emotion with family income or a main effect of family income.

#### Response Time

A three-way Emotion × Condition × Family Income ANCOVA with RT as a dependent variable did not yield any significant interactions or main effects.

### Associations among Family Income, Emotional Response Inhibition, and Internalizing Symptoms

#### Indirect Effect of Family Income on Internalizing Symptoms via an Emotional Response Inhibition Pathway

The indirect effect of family income on child report of inter nalizing symptoms via an emotional response inhibition pathway was tested using Preacher and Hayes’ bootstrapping analysis. Two indirect effect analyses, false alarm rates of each angry and sad “NoGo” distracter face trials as a mediator, were conducted with family income as an independent variable, internalizing symptoms as outcome variables, with IQ and race as covariates in both models.

Bootstrapping analyses revealed a significant *indirect* effect of family income on internalizing symptoms via false alarm rates during angry distracter face trials (indirect effect = -0.17, CIs = -0.504 to -0.002), and via false alarm rates during sad distracter face trials (indirect effect = -0.14, CIs = -0.455 to -0.002). Thus, preliminary evidence suggests that lower income children exhibited greater emotional response inhibition difficulties (i.e., greater false alarm rates to angry and sad “NoGo” distracter faces), which were in turn associated with greater levels of internalizing symptoms.

The bootstrapping analysis was repeated to test an alternative hypothesis (i.e., a potential indirect effect of family income on false alarm rates through an internalizing symptoms pathway). False alarm rates in the context of sad and angry distracter face trials were used as outcome variables, and internalizing symptoms as a mediator. However, the indirect effect of family income was not significant via a false alarm rate pathway during angry or sad “NoGo” distracter face trials.

#### Mediation Analysis using Regression

The mediation model using regression analysis was not significant. There was no significant association between family income and cSDQ internalizing symptoms after controlling for race and IQ, *b* = -0.06, *p* > 0.10. Angry (*b* = 0.23) and sad (*b* = 0.24) false alarm rates and internalizing symptoms were associated at trend levels, controlling for race and IQ, *p*s < 0.1.

#### *Post hoc* Analysis: Child SDQ Depression vs. Anxiety Symptoms

Although, the cSDQ measure does not separate anxiety and depression symptoms, as an exploratory analysis, we repeated the indirect effect test using what we consider as anxiety items (items 2, 4, 5) and depressive items (items 1, 3) of the cSDQ (see Supplementary Materials for item breakdown). Family income was negatively associated with anxiety symptoms, *r*(41) = -0.31, *p* < 0.05, but was not significantly associated with depressive symptoms, *r*(41) = -0.20, *p* > 0.10. Using the calculated anxiety symptom scores, we tested the indirect effect of family income on cSDQ anxiety scores via an emotional response inhibition pathway, with covariates IQ and race. Bootstrapping analyses revealed a significant *indirect* effect of family income on cSDQ anxiety symptoms via false alarm rates during angry distracter face trials (indirect effect = -0.12, CI = -0.35 to -0.01), and via false alarm rates during sad distracter face trials (indirect effect = -0.10, CI = -0.33 to -0.008). Significant indirect effects were not observed when cSDQ depression scores were included in the model in place of cSDQ anxiety scores.

## Discussion

The present study examined the associations between family income, internalizing symptoms, and emotional response inhibition in middle childhood. We further examined the indirect effect of family income on child internalizing symptoms via an emotional response inhibition pathway. We found that lower family income was associated with emotional response inhibition difficulties particularly in the context of task irrelevant angry and sad emotional faces. In addition, greater emotional response inhibition difficulties in the context of angry and sad faces were in turn associated with increased self-reported internalizing symptoms.

### Family Income and Emotional Response Inhibition

Using the Emotion Go/NoGo task, we found that children from lower income homes exhibited emotional response inhibition difficulties in the context of negatively valenced distracters. Lower income was associated with greater false responses to angry and sad distracter “NoGo” faces presented with a series of neutral target “Go” faces. The false alarm rate findings suggest that children from lower income homes were more likely to be influenced by task irrelevant negative emotional information. Failure to inhibit in the context of sad and angry expressions may be due to greater exposure to emotionally relevant stressors in the context of poverty. Our findings are consistent with a growing body of literature that shows the negative effects of early exposure to poverty-related stressors on emotion processing and emotion regulation (Kim et al., 2013; Luby et al., 2013; Javanbakht et al., 2015). Children from impoverished circumstances are more likely to be exposed to chronic psychological stressors, harsh environments, and early adversity (Evans, 2004; Evans et al., 2013). Compared to wealthier children, poor children are more likely to be deprived of supportive environments, experience harsher and less responsive parenting, and receive less optimal care (Wood, 2003; Evans, 2004). Childhood poverty has also been associated with greater daily hassles and stressful life events (e.g., family turmoil/chaos, inadequate housing, diminished resources, interparental aggression) that negatively impact child neurocognitive and socio-emotional development (Evans and English, 2002; Evans et al., 2005; Hanson et al., 2013; Luby et al., 2013). When economic strains are high, parents are also at an increased risk of developing emotional (e.g., depression, anxiety) and behavioral problems (e.g., substance use) and exhibit harsher and less supportive parenting, which in turn have been associated with child emotional problems, including internalizing symptoms (Conger and Donellan, 2007). Thus, poor children are more likely than their middle- and high-income counterparts to experience psychological distress associated with poverty. Exposure to these stressors is in turn associated with neurocognitive deficits and emotional problems (Kim et al., 2013; Luby et al., 2013; Javanbakht et al., 2015), which is consistent with our current findings.

We recognize that some may argue that false responses during angry and sad “NoGo” distracter face trials may instead be due to emotion recognition difficulties associated with poverty (Raver et al., 2015). However, we found that in our sample, lower income children performed with the same accuracy as middle income children when angry and sad faces were the *target* stimuli (i.e., angry and sad target “Go” faces were presented with a series of neutral distracter “NoGo” faces). This finding suggests that lower income children recognized angry and sad facial expressions as well as middle income children.

### Family Income and Fear Recognition

It is important to note that inhibition difficulties were not observed in the context of fearful “NoGo” distracter face trials. Angry faces may signify a direct threat, whereas fearful faces may be more ambiguous and provide less information about the source of threat (Whalen et al., 2001). Thus, angry relative to fearful faces may be more salient and hold more ecological significance to lower income children. Another possible explanation could be that lower income children have greater difficulty recognizing fearful faces, as reflected by the lower percentage of correct responses when fearful faces were the target stimuli. In general, younger children are substantially worse at recognizing fearful expressions relative to other emotional expressions (e.g., happy, angry, sad; Lawrence et al., 2015). It is suggested that fear is a later developing emotion expressed more so by children’s peers than adults, whereas anger is displayed more by adults to young children (Lawrence et al., 2015). Perhaps angry emotional expressions are expressed even more frequently by adults (i.e., parents) in lower income homes (e.g., parental conflict, harsh parenting; Evans, 2004), which could account for its salience relative to fearful expressions. This is consistent with parental conflict and harsh parenting studies (Pollak and Kistler, 2002; Briggs-Gowan et al., 2015; Schermerhorn et al., 2015) that showed aberrant emotion processing of angry information, and over identification of angry expressions relative to other emotions such as fear (Pollak and Kistler, 2002). It is important to note, however, that neuroimaging studies of maternal deprivation found heightened amygdala activity in response to fearful stimuli, suggesting neural hyper-reactivity to fearful information (Tottenham et al., 2011b; Gee et al., 2013). On the other hand, in adults and adolescents, socioeconomic disadvantages have been associated with heightened amygdala responses to angry faces (Gianaros et al., 2008; Muscatell et al., 2012). However, among these studies, few have included both angry and fearful stimuli in the paradigm. Thus, more studies including both fearful and angry stimuli among children who are exposed to poverty and other types of adversity would help to confirm whether poverty may be specifically associated with emotional response inhibition in the context of angry faces or negative emotional information in general. It is also plausible that the lower accuracy we observed during the “Go” fearful trial condition is due to sustained attention problems to fearful emotional stimuli. However, it is difficult to investigate the potential role of sustained attention using this task. To test the potential role of sustained attention, future eye tracking studies that measure attention allocation differences (e.g., sustained attention or attention avoidance) to fearful stimuli between low- and middle- income children may be useful.

### Indirect Effect of Family Income on Internalizing Symptoms through an Emotional Response Inhibition Pathway

Importantly, difficulties in response inhibition in the context of sad and angry faces were further associated with internalizing symptoms. In the current study, children form lower income homes exhibited greater emotional response inhibition difficulties in the context of negative (angry and sad) emotional distracter faces, which in turn were associated with greater levels of internalizing symptoms. This finding suggests that emotional response inhibition difficulties may play a significant role in the development of internalizing symptoms among economically disadvantaged children. Failure to inhibit in the context of negative stimuli may indicate enhanced sensitivity to emotional negative information which may be adaptive in the context of poverty (i.e., better detection of danger cues). However, as our results suggest, repeated and exaggerated reactivity to negative information can also have adverse effects on emotional health (Ellis and Del Giudice, 2014), perhaps one of which is the development of internalizing symptoms.

### Alternative Socioeconomic Disadvantage Variable

Although the current study focused on the effects of poverty exposure on child internalizing symptoms and emotional response inhibition, other types of socioeconomic variables such as parental education have also been associated with offspring outcomes (e.g., cognitive, educational, and emotional outcomes; Roberts et al., 1999; Dubow et al., 2009; Carneiro et al., 2011; Quesnel-Vallee and Taylor, 2012; Park et al., 2013; Sonego et al., 2013; Wirback et al., 2014). In the current study, we examined parental education, but did not find a significant link with emotional response inhibition. The non-significant association may be due to the limited variability in parental education levels in our sample. Thus, studies with a more diverse sample would be important to further examine the overlapping and unique role of family income and parental education on children’s emotional response inhibition abilities and internalizing symptoms.

### Depressive vs. Anxiety Symptoms

As an exploratory analysis, we investigated whether the anxiety or depressive symptoms of the child SDQ were associated with income and emotional response inhibition. The exploratory analysis further suggests that family income was more strongly associated with anxiety symptoms than depressive symptoms, and higher false alarm rates for angry and sad faces were further associated with the child SDQ anxiety scores. This is consistent with previous studies (Tottenham et al., 2010) demonstrating the link between false alarm rates and anxiety symptoms. Given the exploratory nature of the analysis, future studies using more extensive self-report measures such as the Children’s Depression Inventory (CDI; Kovacs, 1992) or the Revised Children’s Manifest Anxiety Scale (RCMAS; Reynolds and Richmond, 1985), or a clinical diagnostic interview could be helpful in further examining the links between emotional response inhibition deficits in the context of specific emotional information and internalizing diagnosis.

### Limitations and Future Studies

The results of the present work should be cautiously interpreted. First, the small sample size makes it difficult to draw conclusions about the relationship between family income and emotional response inhibition. However, the effect size (using partial eta squared) of the main interaction analyses is large ($\eta_{p}^{2}$ > 0.1) especially considering the small sample. Second the cross-sectional design makes it difficult to draw causal inferences and conclusions about the direction of influence of poverty exposure and emotional response inhibition on internalizing symptoms. To address this limitation we examined alternative models and used internalizing symptoms as a possible mediator between family income and emotional response inhibition (angry and sad), however the models did not yield significant findings. The small sample size can also potentially limit external validity and findings may not generalize to the general population and/or clinical samples with depressive and anxiety disorders. In addition, given the differential emotional face processing observed between men and women (McClure, 2000), factors such as gender may also play a role in Emotional Go/NoGo task performance (Schulz et al., 2007), but the small sample size make it difficult to tease out these potential effects. Future studies with larger and more diverse participants are necessary. Furthermore, in the current path analysis, the direct effect of family income on internalizing symptoms was not significant. The non-significant association between family income and internalizing symptoms may in part be due to the small sample size. Studies with larger samples have consistently reported a robust association between childhood poverty and internalizing symptoms (Brooks-Gunn and Duncan, 1997; Murali and Oyebode, 2004; Slopen et al., 2010). Instead, the current study has identified a significant indirect effect of family income on internalizing symptoms via an emotional response inhibition pathway. Studies suggest that a meaningful indirect effect can be observed without a significant relationship between the independent and dependent variable (MacKinnon et al., 2000; MacKinnon et al., 2008; Hayes, 2009; Erisen, 2010). However, it is important to be cautious when interpreting the findings beyond the indirect effect, and future studies with larger samples will be important to establish the full or partial mediating role of emotional response inhibition in the link between family income and internalizing symptoms. Further studies are needed to examine prospective relations among family income, emotional response inhibition, and internalizing symptoms. It is unclear whether the emotional response inhibition difficulties we observed among lower-income children will be associated with later diagnosis of anxiety and depression. In the present study, lower family income was associated with higher levels of self-reported internalizing symptoms. However, we found no association between mother-reported internalizing symptoms and family income. Some suggest that child-report questionnaires may be a more reliable measure since parent-report internalizing instruments can be sensitive to rater bias (Kujipers et al., 2015). However, future research using a clinical diagnostic interview may be helpful in examining the links between emotional response inhibition deficits in the context of specific emotional information and internalizing diagnoses.

## Conclusion

In summary, the current study revealed that middle childhood poverty is related to emotional response inhibition deficits particularly in the context of angry and sad emotional information. These emotional response inhibition deficits were in turn associated with greater self-report internalizing symptoms in middle childhood. By examining impoverished children during an important developmental period, we gain a better understanding of the emotional vulnerabilities of this high-risk group. To our knowledge, this is one of the first studies to explore the socioemotional deficits associated with childhood poverty using an emotional-cognitive control computer task. The current findings inform neuroimaging studies that show heightened amygdala responses to negatively valenced cues in the context of poverty. Future neuroimaging studies may confirm whether heightened neural reactivity to negatively valenced cues may be involved in the emotional response inhibition deficits observed in the context of childhood poverty. Emotional response inhibition deficits may increase risks for internalizing problems, particularly anxiety disorders, among poor children, which is in line with evidence that suggests emotional inhibitory control deficits among clinical mood and anxiety disorders (Ladoucer et al., 2006). One implication for intervention that these findings afford is the potential of training children in emotional response inhibition to help attenuate internalizing symptoms (Raviv and Wadsworth, 2010) among disadvantaged children.

## Author Contributions

All authors of the paper (CC, HB, and PK) have fulfilled the criteria for authorship. The undersigned authors warrant that the material contained in the manuscript represents original work. The authors acquired, analyzed, and interpreted collected data. The final version of the manuscript was approved by the authors and all agreed to be accountable to all aspects of the work.

## Conflict of Interest Statement

The authors declare that the research was conducted in the absence of any commercial or financial relationships that could be construed as a potential conflict of interest.
