# Supplementary material for: Poverty and Internalizing Symptoms: The Indirect Effect of Middle Childhood Poverty on Internalizing Symptoms via an Emotional Response Inhibition Pathway
Source: Front Psychol. 2016 Aug 17;7:1242. doi: 10.3389/fpsyg.2016.01242 (PMC4987327; doi:10.3389/fpsyg.2016.01242)
Supplement: Supplementary file 1 [file Data_Sheet_1.DOCX]

**Supplementary Material**

**Emotion X Condition ANOVA**

A two-way repeated measures ANOVA was conducted for the dependent measure of false alarm rate [Emotion (happy, fearful, angry, and sad) and Condition (Go and NoGo) as within-subjects factors]. Significant interactions were decomposed using post-hoc t-tests.

**Results**

In the Tottenham et al. study, average accuracy level was 75 percent for negatively valenced stimuli and 82 percent for positively valenced stimuli in children ages 5-12 (Tottenham, Hare, & Casey, 2011), which is similar to our accuracy findings. Furthermore, when a separate analysis was conducted without the income variable [Condition (Go and NoGo) x Emotion (angry, fearful, happy, sad) on the dependent variable of false alarm rates], a main effect of Condition, *F*(1, 42) = 4.70, *p* < 0.05, η²p = 0.10, and Emotion, *F*(1, 42) = 29.06, *p* < 0.001, η²p = 0.41 was observed. Posthoc analyses suggest that regardless of emotion, false alarm rates were significantly higher during the NoGo condition, or when Neutral target faces were embedded within a series of emotional faces, *t*(42)=-2.17, *p* < 0.05. Across conditions, the greatest false alarm rate was for sad [mean (SD) = 0.53 (0.20); *t* (relative to angry) = 5.16, *p* < 0.001; *t* (relative to fear) = 6.86, *p* < 0.001; *t* (relative to happy) = 7.61, *p* < 0.001], followed by angry [mean (SD) = 0.40 (0.18); *t* (relative to fear) = 1.33, *p* = 0.19; *t* (relative to sad) = -5.16, *p* < 0.001]; *t* (relative to happy) = 4.03, *p* < 0.001], and then fear [mean (SD) = 0.37 (0.19)]; *t* (relative to happy) = 2.55, p < 0.05], and happy [mean (SD) = 0.31 (0.17)], which mirror the Tottenham et al. findings.

**Strengths and Difficulties Questionnaire Items:**

The items of the emotional subscale of the cSDQ are as follows:

Item 1 (somatic symptoms): I get a lot of headaches, stomach-aches, or sickness

Item 2 (worries): I worry a lot.

Item 3 (unhappy): I am often unhappy, depressed, or tearful

Item 4 (clingy, nervous in new situations): I am nervous in new situations. I easily lose confidence.

Item 5 (afraid many fears): I have many fears. I am easily scared.

As an exploratory analysis, we separated what we consider as anxiety items (items 2, 4, 5) and depressive items (items 1, 3).
